# Isolated limb perfusion with actinomycin D and TNF-alpha results in improved tumour response in soft-tissue sarcoma-bearing rats but is accompanied by severe local toxicity

**DOI:** 10.1038/sj.bjc.6600169

**Published:** 2002-04-08

**Authors:** A L B Seynhaeve, J H W de Wilt, S T van Tiel, A M M Eggermont, T L M ten Hagen

**Affiliations:** Department of Surgical Oncology, University Hospital Rotterdam Dijkzigt/Daniel den Hoed Cancer Center, Rotterdam, the Netherlands

**Keywords:** tumour necrosis factor alpha, actinomycin D, isolated limb perfusion, rats

## Abstract

Previously we demonstrated that addition of Tumour Necrosis Factor-α to melphalan or doxorubicin in a so-called isolated limb perfusion results in synergistic antitumour responses of sarcomas in both animal models and patients. Yet, 20 to 30% of the treated tumours do not respond. Therefore agents that synergise with tumour necrosis factor alpha must be investigated. Actinomycin D is used in combination with melphalan in isolated limb perfusion in the treatment of patients with melanoma in-transit metastases and is well known to augment tumour cell sensitivity towards tumour necrosis factor alpha *in vitro*. Both agents are very toxic, which limits their systemic use. Their applicability may therefore be tested in the isolated limb perfusion setting, by which the tumours can be exposed to high concentrations in the absence of systemic exposure. To study the beneficial effect of the combination *in vivo*, BN-175 soft tissue sarcoma-bearing rats were perfused with various concentrations of actinomycin D and tumour necrosis factor alpha. When used alone the drugs had only little effect on the tumour. Only when actinomycin D and tumour necrosis factor alpha were combined a tumour response was achieved. However, these responses were accompanied by severe, dose limiting, local toxicity such as destruction of the muscle tissue and massive oedema. Our results show that isolated limb perfusion with actinomycin D in combination with tumour necrosis factor alpha leads to a synergistic anti-tumour response but also to idiosyncratic locoregional toxicity to the normal tissues. Actinomycin D, in combination with tumour necrosis factor alpha, should not be explored in the clinical setting because of this. The standard approach in the clinic remains isolated limb perfusion with tumour necrosis factor alpha in combination with melphalan.

*British Journal of Cancer* (2002) **86**, 1174–1179. DOI: 10.1038/sj/bjc/6600169 www.bjcancer.com

© 2002 Cancer Research UK

## 

In 1958 Creech *et al* (1958) described the application of an isolated limb perfusion (ILP) for the treatment of a patient with a melanoma in-transit metastases who refused amputation. Perfusions with melphalan in these patients results in fairly good response rates in contrast to patients with locally advanced extremity soft tissue sarcomas (STS) (Eggermont, 1996a). The addition of tumour necrosis factor-alpha (TNF) has dramatically changed the efficacy of the procedure in patients with extremity STS. Eggermont *et al* (1996b,c) reported that in patients with advanced STS an ILP with TNF + melphalan with Interferon-gamma or without interferon-gamma improved tumour response rates to above 80% resulting in a limb salvage in over 70% of the patients destined otherwise to undergo an amputation. The results of the multicenter studies in Europe eventually lead to the approval of TNF for this indication in Europe (Eggermont *et al*, 1999). Despite these good results, still 20 to 30% of these patients do not respond to this therapy and other agents that can further improve this treatment must be identified.

In rat osteo- and soft tissue sarcoma ILP models we were able to mimic the clinical ILP with comparable response type and rate (Manusama *et al*, 1996). In this setting new drug combinations can be tested rapidly. To improve response rates doxorubicin has been used instead of melphalan. However, ILP with doxorubicin in combination with TNF appeared less effective than melphalan with TNF. Response rates were respectively 54 and 70% (de Wilt *et al*, 1999; Van Der Veen *et al*, 2000).

Actinomycin D is an anticancer antibiotic that has been used in combination with melphalan in the ILP setting in the treatment of patients with melanoma in-transit metastases confined to the extremities. It is an interesting drug to evaluate in combination with TNF because incubation of tumour cells with actinomycin D has been shown to increase their sensitivity to the effects of TNF (Alexander *et al*, 1987). Administration of TNF and actinomycin D in mice delayed the growth of several tumours significantly, but application was limited due to the severe toxic effects (Mosende *et al*, 1977). Patients with osteogenic sarcoma have been treated with actinomycin D in combination with bleomycin and cyclophosphamide. This treatment was associated with severe nausea and anorexia (Lasek *et al*, 1996). With an ILP a high drug concentration can be administered with minimal systemic toxicity due to negligible leakage to the rest of the body (Lienard *et al*, 1992). In this way, melphalan has been used for many years as a single drug treatment with a local concentration 15–20 times higher than can be achieved by systemic treatment (Benckhuijsen *et al*, 1988).

In the presented study we performed ILP with the combination of actinomycin D and TNF to study their potential of inducing a tumour response. *In vitro* studies were undertaken to examine the direct effect of the agents on tumour cells.

## MATERIALS AND METHODS

### Animals

Male inbred BN rats, weighing 250–300 g, obtained from Harlan-CPB (Austerlitz, the Netherlands) were used for isolated limb perfusions. Rats were fed a standard laboratory diet ad libitum (Hope Farms Woerden, the Netherlands) and were housed under standard conditions. The experimental protocols adhered to the rules outlined in the ‘Dutch Animal Experimentation Act’ (1977) and the published ‘Guidelines of the UKCCCR for the welfare of animals in experimental Neoplasia’ (UKCCCR, 1998). The protocol was approved by the committee on Animal Research of the Erasmus University Rotterdam, the Netherlands.

### Tumour model

The rapidly growing and metastasising BN-175 soft tissue sarcoma, which is transplantable to the BN rat, was used. Fragments of 2–3 mm were implanted subcutaneously in the right hind limb, just above the ankle. Perfusion was performed at a tumour diameter of 13±2 mm, approximately 7 days after implantation. Tumour growth was recorded by calliper measurements and the volume was calculated with the formula 0.4 (A^2^×B), where B stands for the largest diameter of the tumour and A the diameter perpendicular to B.

### Drugs

Recombinant human TNF (rHuTNF) was provided by Boehringer (Boehringer Ingelheim GmbH, Austria) with a specific activity of 5.8×10^7^ U mg^−1^ as determined in the murine L-M cell assay. Endotoxin levels were <1.25 units (EU) per mg protein. TNF concentrations used were 50 μg in 5 ml perfusate.

Actinomycin D (Sigma, the Netherlands) was diluted in phosphate buffered saline to a concentration of 2 mg ml^−1^. Concentrations used were 5 and 10 μg in 5 ml perfusate.

Melphalan (Alkeran, Wellcome, Beckenham, UK) was diluted in phosphate buffered saline to a concentration of 2 mg ml^−1^. Concentrations used were 40 μg in 5 ml perfusate.

### *In vitro* assessment of anti-tumour activity

Cells isolated from a BN-175 soft tissue sarcoma were maintained in cell culture for a maximum of 20 passages in RPMI supplemented with 10% foetal bovine serum and L-glutamine. Media and supplements were obtained from Life Technologies, the Netherlands.

BN soft tissue sarcoma cells were added in 100 μl aliquots to 96-well plates at a final concentration of 10^4^ cells per well and allowed to grow as a monolayer. Actinomycin D and rHuTNF, diluted in RPMI supplement with 10% foetal bovine serum and L-glutamine, were added to the wells and allowed to incubate for 3 days. The range of final drug concentration in the well was 0.05–100 ng ml^−1^ for actinomycin D and 0–10 000 ng ml^−1^ for rHuTNF. As a control the TNF sensitive cell line WEHI-164 was used. The cells were incubated in the presence of a concentration of 0.05–100 ng ml^−1^ actinomycin D and 0–1 ng ml^−1^ TNF, diluted in RPMI supplement with 10% foetal bovine serum and L-glutamine. The sulphorhodamine B (SRB) protein stain assay was used according to the method of Skehan *et al* (1990). Briefly, cells were washed with phosphate buffered saline (PBS), incubated with 10% trichloric acetic acid in distilled water (1 h, 4°C) and washed again in distilled water. Cells were then stained for 15 to 30 min with SRB (Sigma, St. Louis, MO, USA), washed with 1% acetic acid in distilled water and allowed to dry. Protein bound SRB was dissolved in 10 mM Tris buffer, pH 9.4 and absorption was measured at 540 nm. Tumour growth was calculated using the formula: percentage tumour growth=(test well/control well) ×100%.

### Isolated Limb Perfusion (ILP) model

The perfusion technique was performed as described previously (de Wilt *et al*, 1999). Briefly, animals were anaesthetised with Ketalin (Apharmo, Duiven, the Netherlands) and Xylazin (Bayer B.V., Mijdrecht, the Netherlands). To prevent coagulation 50 IU of heparin was injected intravenously. To keep the rat's hind limb at a constant temperature of 38–39°C, a warm water mattress was applied. Temperature was measured with a temperature probe on the skin covering the tumour. The femoral artery and vein were cannulated with silastic tubing (0.012 in inner diameter (ID), 0.025 in outer diameter (OD); 0.025 in ID, 0.047 in OD respectively, Dow Corning, MI, USA). Collaterals were occluded by a groin tourniquet, and isolation time started when the tourniquet was tightened. An oxygenation reservoir and a roller pump were included into the circuit. The perfusion solution was 5 ml Haemaccel (Behring Pharma, Amsterdam, the Netherlands). The cytotoxic drugs with or without TNF were added as boluses to the oxygenation reservoir. A roller pump (Watson Marlow, Falmouth, UK; type 505 U) recirculated the perfusate at a flow rate of 2.4 ml min^−1^. A washout with 5 ml oxygenated Haemaccel was performed at the end of the perfusion.

Tumour growth was daily recorded by calliper measurement. Tumour volume was calculated as 0.4 (A^2^B), where B represents the longest diameter and A the diameter perpendicular to B.

### Assessment of limb function

Limb function was a clinical observation in which the rat's ability to walk and stand on the perfused limb was scored 1 day after ILP and is an identification for the toxicity of the used chemicals. On this scale a severe impaired function (grade 0) means that the rat drags its hind limb without any function; a slightly impaired function (grade 1) means the rat does not use its hind limb in a usual matter, but stands on it when rising; an intact function of the hind limb (grade 2) means a normal walking pattern.

Oedema was scored as no formation of oedema (−), or oedema resulting in leg diameter increase of 1.5-fold or more (+).

### Histology

The day after perfusion with actinomycin D alone or in combination with TNF tumour and muscle tissue of the perfused limb was removed fixed for 24 h in 4% formaldehyde and embedded in paraffin. Muscle from the non-perfused limb was also obtained, fixed and embedded. Tissue sections of 4 μm were cut and stained with haematoxylin and eosin, followed by examination using a Leica DM-RXA.

### Statistical analysis

Mann–Whitney *U*-test was used to compare tumour volumes. Calculations were performed on a personal computer using GraphPad Prism v3.0 and SPSS v8.0 for Windows 98.

## RESULTS

### *In vitro* assessment of anti-tumour activity

**Figure 1:**
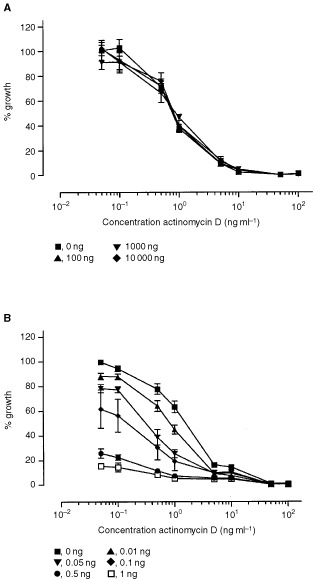
*In vitro* growth of BN-175 sarcoma cells (**A**) as a function of the actinomycin D concentration in combination with 0 ng, 100 ng, 1000 ng and 10 000 ng TNF per ml. The mean of seven individual experiments performed in duplicate are shown (±s.e.m.). And the *in vitro* growth of WEHI-164 murine cells (**B**) as a function of the actinomycin D concentration in combination with 0 ng, 0.01 ng, 0.05 ng, 0.1 ng, 0.5 ng and 1 ng TNF per ml. The mean of seven individual experiments performed in duplicate are shown (±s.e.m.).

Previously, we demonstrated the lack of synergy between melphalan and TNF *in vitro* (Manusama *et al*, 1996). To investigate the effect of actinomycin D and TNF directly on the BN-175 tumour cells a bioassay was performed. Exposure of the BN-175 sarcoma cells to actinomycin D resulted in a dose related cytotoxicity with an IC_50_ of 1 ng ml^−1^ (Figure 1A

). When used alone TNF had no cytotoxic or cytostatic effect on this tumour cell line. Addition of TNF to actinomycin D did not alter the IC_50_ of actinomycin D, indicating lack of any synergy between the two drugs on this cell line. Incubation of the TNF sensitive cell line WEHI-164 resulted in synergy when the cells were treated with a combination actinomycin D and TNF (Figure 1B).

### *In vivo* tumour response to actinomycin D and TNFafter ILP

We evaluated the anti-tumour effect of actinomycin D and melphalan with or without TNF after an isolated limb perfusion. BN-175 soft tissue sarcoma-bearing rats were perfused with different concentrations of actinomycin D or melphalan with or without TNF.

**Figure 2:**
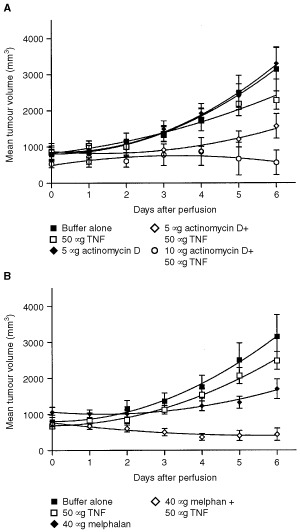
Growth curves of subcutaneous implanted BN-175 sarcoma (**A**) after isolated limb perfusion with buffer alone, 50 μg TNF, 5 μg actinomycin D, 5 μg actinomycin D combined with 50 μg TNF and 10 μg actinomycin D combined with 50 μg TNF and (**B**) after isolated limb perfusion with buffer alone, 50 μg TNF, 40 μg melphalan and 40 μg melphalan combined with 50μg TNF. Mean (±s.e.m.) of tumour volumes are shown.

Perfusions with Haemaccel (sham), or with 50 μg TNF or 5 μg actinomycin D alone, resulted in progressive disease in all animals (Figure 2A

). Addition of 50 μg TNF to ILP with 5 μg actinomycin D not only resulted in inhibition of the tumour growth, but also in tumour shrinkage. At 5 days after ILP a significant difference in the mean tumour volume was observed as compared to sham perfusions (*P*<0.05), TNF perfusions alone (*P*<0.05) and actinomycin D perfusions alone (*P*<0.05). The perfusions with a combination of 5 μg actinomycin D and 50 μg TNF resulted in a response rate of 40% (data not shown). When perfusions were performed with a higher concentration of actinomycin D a further increase in tumour response was observed. However due to the severe toxicity with this concentration no further experiments were performed. Perfusions with 40 μg melphalan and 50 μg TNF resulted also in major tumour shrinkage. A significant difference was observed as compared to sham perfusions, TNF perfusions alone and melphalan perfusions alone (*P*<0.05) (Figure 2B). Perfusions with 40 μg melphalan and 50 μg TNF resulted in a response rate of 70% (data not shown), conform our experience as published in various extensive series of ILP with melphalan and TNF (Manusama *et al*, 1996; de Wilt *et al*, 1999).

### Hind limb function and local toxicity

Shortly after ILP with actinomycin D in combination with TNF severe local toxicity was observed (Table 1

**Table 1:**
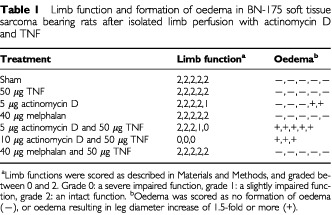
Limb function and formation of oedema in BN-175 soft tissue sarcoma bearing rats after isolated limb perfusion with actinomycin D and TNF

). Sham perfusions and perfusions performed with TNF alone resulted in no loss of the limb function. Some toxic side effects were observed in the rats that were perfused with actinomycin D alone. However, after the addition of TNF to perfusion with actinomycin D the limb function dramatically deteriorated and strong formation of oedema was observed. After perfusion with melphalan with or without TNF no loss of limb function or oedema formation was observed. Observation of the limb were made just before and 1 day after perfusion with actinomycin D and TNF. Before perfusion the rats showed a tumour in the right hind limb of approximately 1000 mm^3^. Limb function at this stage was normal and no oedema could be observed. One day after perfusion with actinomycin D in combination with TNF the rats limb was severely swollen with oedema and the remaining tumour was barely visible. Perfusion with buffer or TNF alone had no effect on formation of oedema, whereas perfusion with actinomycin D had only a marginal effect.

**Figure 3:**
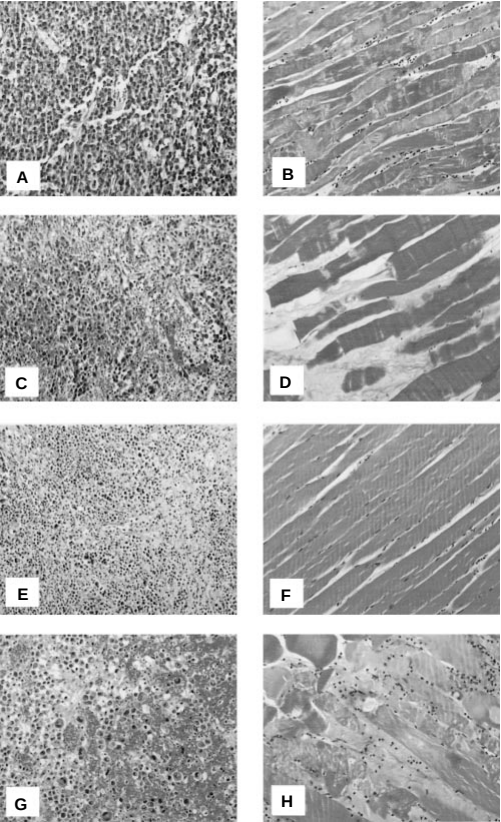
Tissue sections of BN-175 tumour and muscle, haematoxylin and eosin stained, 1 day after isolated perfusion (ILP). Histologic overview of BN-175 tumour (**A**) and muscle (**B**) of the rat limb 1 day after sham ILP. Histologic sections of tumour tissue (**C**) and muscle (**D**) after ILP with actinomycin D, tumour tissue (**E**) and muscle (**F**) after ILP with TNF, tumour (**G**) and muscle (**H**) after ILP with actinomycin D plus TNF. Original magnification of all sections was 16×.

Tumour tissues as well as healthy tissues (muscle, skin) were examined histopathologically. After sham perfusion some scattered cell necrosis in tumours could be observed, correlating with previous results (Figure 3A

). In the muscle tissue from the sham perfused limb, the skeletal muscular fibre and the peripheral nuclei could be observed in a normal pattern. The fibres were intact and no destruction of the tissue was found (Figure 3B). Rats treated with actinomycin D alone showed massive infiltration of red blood cells and haemorrhagic necrosis in the tumour 1 day after perfusion (Figure 3C). Histological slides taken from the muscle of the limb perfused with only actinomycin D showed destruction of some of the muscle cells (Figure 3D). One day after perfusion with TNF alone haemorrhagic necrosis in tumour was observed. However the infiltration of red blood cells was not as pronounced as after a perfusion with actinomycin D (Figure 3E). Rats treated with TNF alone showed no damage to the muscle (Figure 3F). Rats treated with a combination actinomycin D and TNF showed again massive infiltration of red blood cells and haemorrhagic necrosis in tumour (Figure 3G). Treatment with a combination of actinomycin D plus TNF showed massive destruction of the muscle tissue. Cells were torn apart and the nuclei were released in the surrounding muscle tissue (Figure 3H).

## DISCUSSION

Previously we have demonstrated that local treatment of advanced tumours with a combination of melphalan and TNF results in high response rates. We developed a rat tumour model and showed that results comparable to the clinic can be obtained regarding response rate and type of response (Manusama *et al*, 1996; de Wilt *et al*, 1999; Van Der Veen *et al*, 2000). We used this model to evaluate other drug combinations to improve tumour responses or to diminish toxic side effects. We recently demonstrated that addition of L-NAME to perfusions with melphalan with or without TNF enhanced antitumour effects (de Wilt *et al*, 2000a). A mutant of TNF, TNF-SAM2, was also investigated in this model and demonstrated similar efficacy as observed with TNF (de Wilt *et al*, 2000b). In the BN-175 soft tissue sarcoma rat ILP model the usefulness of actinomycin D was evaluated in correlation to response and toxicity. Actinomycin D is of particular interest both because of its use in the ILP setting in the clinic and because of its interactions with TNF *in vitro*. Treatment with melphalan in combination with actinomycin D has been reported, but these studies showed no significant difference in response between treatment with the single agent melphalan and the combination therapy (Park *et al*, 1980). In previous studies with TNF and actinomycin D synergistic effects *in vitro* were seen (Mosende *et al*, 1977; Aoki *et al*, 1998). Especially the TNF sensitive tumour cell line WEHI-164 responded in a synergistic fashion to the combination of actinomycin D and TNF. The soft tissue sarcoma BN-175 used in this study was found to be susceptible to actinomycin D, but addition of TNF did not result in a more cytotoxic effect on these tumour cells. These results correlate with *in vitro* studies using melphalan or doxorubicin in combination with TNF on several rat tumour cell lines as colon carcinomas and osteosarcomas (van Der Veen *et al*, 2001). However, *in vivo* combination of melphalan as well as doxorubicin with TNF did result in strong synergy both in patients and animal models (Lejeune *et al*, 1995; Eggermont *et al*, 1996b,c, 1997, 1999; Manusama *et al*, 1996; Di Filippo *et al*, 1999; Van Der Veen *et al*, 2000). We speculated that this is due to the dual targeting of the combination. Whereas the cytotoxic drugs preferentially target tumour cells, TNF mainly affects the tumour associated vasculature, possibly resulting in destruction of the vessels and augmented accumulation of the drugs in the tumour tissue (Eggermont *et al*, 1997; de Wilt *et al*, 2000c; ten Hagen *et al*, 2000; Van Der Veen *et al*, 2000).

In the present study an isolated limb perfusion with TNF, actinomycin D or a combination in BN-175 soft tissue sarcoma bearing rats was performed to investigate the tumour response to these treatments. We demonstrated that the combination of actinomycin D with TNF was much more effective than ILP with the single agents. However, the response rate was less pronounced compared to previously described perfusions with melphalan or doxorubicin in combination with TNF.

Application of ILP, with leakage below 5%, allows high local dosages of cytotoxic agents without the systemic side-effects (Benckhuijsen *et al*, 1988; Buckley *et al*, 1989; Vrouenraets *et al*, 1999). This means that local toxicity is the limiting factor. In our study severe toxicity was not observed with actinomycin D or TNF when used as single agents. However, severe toxicity occurred in the rats that underwent an ILP with the combination therapy. This toxicity is only local and consists of permanent loss in limb function and massive oedema formation in the perfused limb.

In conclusion, isolated limb perfusion with actinomycin D and TNF results in a tumour response of 40% which is not better than the standard ILP with melphalan plus TNF. Secondly, this therapy was accompanied by dramatic local toxicity, which increased with higher dosages, whereas response rate improved only marginally. Massive destruction of the muscle tissue was observed and as a result a loss of limb function, indicating that isolated limb perfusion with actinomycin D in combination with TNF should not be explored in the clinical setting.
